# Nano-delivery of miRNA inhibiting CENPF combined with cisplatin for bladder cancer treatment†

**DOI:** 10.1039/d5ra01513h

**Published:** 2025-06-13

**Authors:** Ruixiang Song, Xin Chen, Zhensheng Zhang, Huiqing Wang, Guanghua Chen, Jinshan Xu, Shuxiong Zeng, Wentao Zhang, Xudong Yao

**Affiliations:** a Department of Urology, Shanghai Tenth People's Hospital, Tongji University Shanghai 200072 China; b Department of Urology, Changhai Hospital, Naval Medical University Shanghai 200433 China; c Department of Urology, Zhejiang Qiushi Cardiovascular Hospital Hangzhou 310011 China; d Urologic Cancer Institute, School of Medicine, Tongji University Shanghai 200072 China

## Abstract

Bladder cancer (BCa) presents a substantial global health burden, with high rates of recurrence and metastasis that limit the effectiveness of current therapies. New therapeutic strategies are urgently needed. This study introduces a novel nanotherapeutic approach utilizing polydopamine (PDA) nanoparticles to co-deliver cisplatin and miR-205-5p for BCa treatment. Combination therapy reduces the dose-dependent toxicity of cisplatin while enhancing tumor cell cytotoxicity. miR-205-5p targets centromere protein F (CENPF), a key regulator of cancer progression. Overexpression of CENPF in BCa correlates with poor prognosis, and miR-205-5p-mediated suppression of CENPF expression inhibits tumor growth. The PDA-based system combines the DNA-damaging effects of cisplatin with the gene-silencing properties of miR-205-5p, resulting in synergistic antitumor activity. This multimodal strategy enhances therapeutic precision and efficacy, providing a promising solution for BCa treatment with significant clinical potential.

## Abstract 2

Bladder cancer (BCa) presents a substantial global health burden, with high rates of recurrence and metastasis that limit the effectiveness of current therapies.

## Graphical abstract

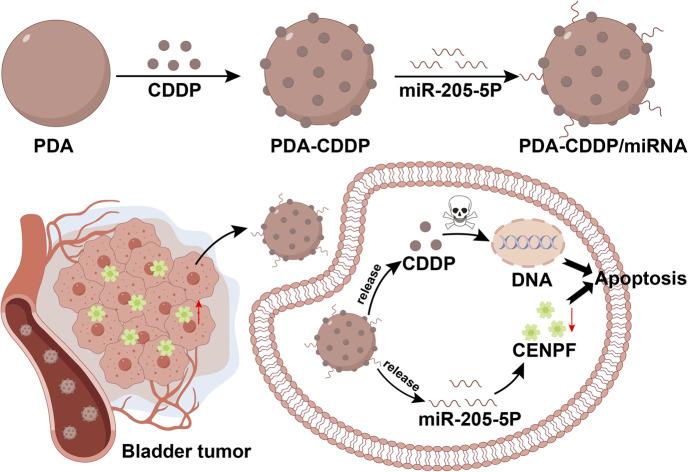

## 1. Introduction

Bladder cancer (BCa) is a major public health concern, representing the most common malignancy of the urinary system.^1^ Over 430 000 individuals are diagnosed with BCa annually, and nearly 170 000 succumb to the disease, imposing a considerable societal burden.^2^ The high recurrence rate and frequent metastasis, particularly in advanced stages, limit the effectiveness of current therapies in enhancing patient survival and quality of life.

Treatment strategies for BCa include surgery, chemotherapy, immunotherapy, and targeted therapies. For non-muscle-invasive bladder cancer (NMIBC), transurethral resection of bladder tumor (TURBT) is commonly followed by intravesical chemotherapy (*e.g.*, cisplatin) or immunotherapy (*e.g.*, Bacillus Calmette–Guérin).^3^ In contrast, muscle-invasive bladder cancer (MIBC) often requires radical cystectomy, typically accompanied by chemotherapy.^4^ Recent breakthroughs have introduced immune checkpoint inhibitors, such as PD-1/PD-L1 antibodies, providing new therapeutic options for BCa patients.^5,6^ However, the response rates to these treatments remain suboptimal, and they may be associated with severe adverse effects. Despite these innovations, cisplatin-based neoadjuvant chemotherapy (NAC) continues to be a cornerstone of perioperative management.^7^ Cisplatin induces DNA crosslinking, impeding DNA replication and promoting cell death, and remains a standard treatment in contemporary clinical guidelines.^8–11^ However, its nephrotoxicity and neurotoxicity limit its use at higher doses,^12–15^ underscoring the need for novel therapies that target molecular pathways in BCa with greater precision.

Centromere protein F (CENPF) is a key player in cell division and belongs to the centromere protein family.^16^ It is critical for chromosome attachment and segregation during mitosis, particularly in the G2/M phase, by regulating microtubule dynamics.^17^ Overexpression of CENPF has been linked to increased proliferation, migration, and invasiveness in several cancers.^18–22^ For example, CENPF contributes to gastric cancer metastasis and angiogenesis *via* the FAK/MAPK and epithelial–mesenchymal transition pathways.^23^ In papillary thyroid cancer, elevated CENPF expression promotes disease progression.^24^ Furthermore, in breast cancer, LncRNA MCM3AP-AS1 accelerates tumor growth by modulating the miR-28-5p/CENPF axis.^25^ In adrenocortical carcinoma, the CENPF/CDK1 signaling pathway regulates the G2/M-phase cell cycle, promoting progression.^26^ Knockdown of CENPF has been shown to inhibit lung adenocarcinoma progression *via* the ERβ2/5 pathway.^27^ Additionally, LINC00536 knockdown suppresses breast cancer growth by indirectly inhibiting CENPF expression.^28^ Thus, CENPF is a promising therapeutic target. In BCa, its overexpression correlates with poor clinical prognosis, with studies indicating that CENPF levels are significantly higher in BCa cells compared to normal bladder tissue.^29^ Inhibition of CENPF has shown efficacy in suppressing cancer cell proliferation, migration, and invasion, highlighting its potential clinical significance. However, research focusing on CENPF targeting in BCa remains limited, indicating the need for further exploration of its therapeutic potential.

**Fig. 1:**
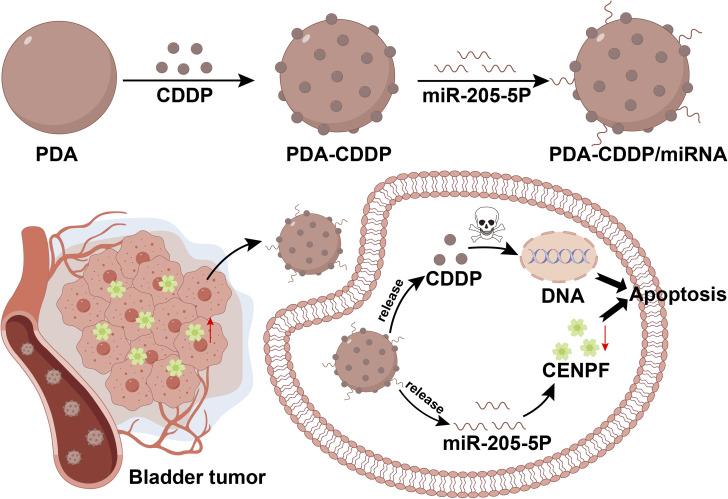
Schematic illustration of the synthesis and therapeutic mechanism of PDA–CDDP/miRNA nanocomplex.

In this study, we developed an innovative therapeutic approach that integrates polydopamine (PDA) nanoparticles with cisplatin and miR-205-5p for enhanced bladder cancer (BCa) treatment (Fig. 1). The PDA nanoparticles, selected for their exceptional biocompatibility and superior drug-loading capacity,^30–33^ serve as a versatile delivery platform for both cisplatin and miR-205-5p, the latter specifically targeting CENPF expression. In this study, we propose an innovative therapeutic strategy that combines polydopamine (PDA) nanoparticles with cisplatin and miR-205-5p for enhanced BCa treatment (Fig. 1). The PDA nanoparticles, chosen for their excellent biocompatibility and high drug-loading capacity, provide a versatile platform for co-delivering cisplatin and miR-205-5p, the latter specifically targeting CENPF expression. This multimodal approach exploits the synergistic effects of cisplatin's DNA crosslinking and miR-205-5p's gene-silencing capability to downregulate CENPF, disrupting key processes in tumor cell division and proliferation. By integrating chemotherapy and gene therapy, this PDA-based nanocarrier system offers improved targeting and enhanced therapeutic efficacy, holding significant promise for clinical translation.

## 2. Materials and methods

### 2.1 Materials

The following reagents were used in this study: dopamine hydrochloride and ammonium hydroxide were purchased from Sigma-Aldrich (St. Louis, MO, USA); cisplatin was obtained from MedChemExpress (Monmouth Junction, NJ, USA); *o*-phenylenediamine (OPD) and fluorescein isothiocyanate (FITC) were acquired from Aladdin Biochemical Technology Co., Ltd (Shanghai, China); Calcein-AM and propidium iodide (PI) were procured from Beyotime Biotechnology (Shanghai, China). All chemicals were of analytical grade and used without further purification.

### 2.2 Preparation of PDA–CDDP

In a 250 mL round-bottom flask, 2 mL of ammonia solution (28–30%), 40 mL of anhydrous ethanol, and 90 mL of ultrapure water were mixed and stirred at room temperature for 30 minutes. Dopamine hydrochloride solution (0.05 g mL^−1^) was then rapidly added. The reaction was allowed to proceed for 24 hours under open-air conditions. Following this, the mixture was dialyzed for 24 hours to obtain polydopamine nanoparticles (PDA NPs). For PDA–CDDP preparation, different concentrations of PDA and cisplatin (CDDP) in DMF were mixed at specific ratios and incubated for 12 hours in the dark at room temperature. The resulting PDA@CDDP complex was then ultrafiltered to remove unbound CDDP.

### 2.3 Preparation of PDA–CDDP/miRNA

Excess miRNA (miR-205-5P, 3′-GUCUGAGGCCACCUUACUUCCU-5′) was added to the PDA–CDDP complex and incubated for 4 hours to enable electrostatic adsorption. The resulting PDA–CDDP/miRNA nanoparticles were purified by ultrafiltration and washed with PBS to remove unbound miRNA. The purified nanoparticles were stored at 4 °C for subsequent use.

### 2.4 Detection of CDDP

A standard cisplatin solution was prepared by dissolving 1 mg of cisplatin in 1 mL of DMF to yield a 1 mg mL^−1^ solution. The solution was then diluted to concentrations of 2.5, 3.125, 5, 6.25, 12.5, and 20 μg mL^−1^. These diluted solutions were mixed with an equal volume of 1.2 mg per mL *o*-phenylenediamine in DMF and heated in an oil bath at 100 °C for 10 minutes. The absorbance of the filtrate was measured at 703 nm. CDDP concentrations were determined using a standard curve.

### 2.5 Stability assessment

The stability of the PDA–CDDP/miRNA nanoparticles was evaluated by dispersing them in a 5% FBS solution. Particle size was measured at 2, 4, 6, 8, 10, 12, and 24 hours to monitor changes over time.

### 2.6 CDDP release study

To assess the drug release behavior, 2 mL of PDA–CDDP/miRNA system was sealed in a dialysis bag and placed in a centrifuge tube containing 10 mL PBS buffer at pH 7.4 and 5.0. The tubes were shaken at 37 °C and 150 rpm. Samples were collected at 2-hour intervals (with further collection every 5 hours after 10 hours). Absorbance was measured at 703 nm, and the CDDP release was quantified using the standard curve.

### 2.7 Fluorescent labeling

Fluorescein isothiocyanate (FITC) was used to label PDA nanoparticles. FITC's isothiocyanate group reacted with free amino groups on the PDA surface, forming carbamide bonds. This process resulted in FITC-labeled fluorescent nanoparticles.

### 2.8 Cell culture

5637 cells (Invitrogen, Carlsbad, USA) were cultured in RPMI 1640 medium (Hyclone), supplemented with 1% penicillin and streptomycin (Hyclone), and 10% fetal bovine serum (FBS; Gibco) in a humidified incubator at 37 °C with 5% CO_2_.

### 2.9 CCK-8 assay

5637 cells were seeded in 96-well plates and incubated for 24 hours to allow attachment. After replacing the medium with fresh culture containing different concentrations of test agents, cells were cultured for a specified duration. CCK-8 solution (10 μL) was added to each well, followed by incubation for 1.5 hours at 37 °C. The absorbance at 450 nm was measured using a microplate reader, and cell viability was calculated by comparing the absorbance values of the treatment and control groups.

### 2.10 Cellular uptake of PDA–CDDP, PDA–miRNA, and PDA–CDDP/miRNA by 5637 cells

FITC-labeled PDA–CDDP, PDA–miRNA, and PDA–CDDP/miRNA were incubated with 5637 cells for different durations. After washing and fixation, cell nuclei were stained with DAPI. Cellular uptake was visualized using confocal microscopy.

### 2.11 Live/dead staining

5637 cells were seeded into 96-well plates and cultured for 24 hours. The medium was replaced with fresh culture containing different concentrations of the test agents, and cells were incubated for an additional 24 hours. Live/dead staining solution was prepared by mixing Calcein-AM and PI at a specified ratio. Calcein-AM labeled live cells with green fluorescence, and PI labeled dead cells with red fluorescence. The staining solution was added, followed by incubation at 37 °C in the dark for 15–30 minutes. Live and dead cells were observed under a fluorescence microscope.

### 2.12 Animal model

All animal experiments were conducted according to ethical guidelines approved by the Animal Care Ethics Committee of Shanghai Tenth People's Hospital, Tongji University School of Medicine (Approval Number: SHDSYY-2019-3028). Female BALB/c nude mice (5 weeks old) were obtained from Shanghai Jihui Laboratory Animal Care Co., Ltd. Tumor models were established by subcutaneously injecting 100 μL of a cell suspension (approximately 1 × 10^6^ cells) into the dorsal region of the mice. Mice were monitored for tumor growth, and tumor dimensions (length and width) were measured using a caliper. Tumor volume was calculated, and when tumors reached the experimental endpoint, mice were euthanized, and tumors were harvested for further analysis.

### 2.13 Statistical analyses

All the statistical analyses were conducted by GraphPad Prism 9 (GraphPad Software Inc., San Diego CA). The sample number for each group was ≥3, and numerical data were reported as mean ± SD *in vitro* and mean ± SEM *in vivo*. *p* values were considered to be statistically significant at *p < 0.05, **p < 0.01, and ***p < 0.001.

## 3. Results and discussion

### 3.1 Synthesis and characterization of PDA–CDDP/miRNA

**Fig. 2:**
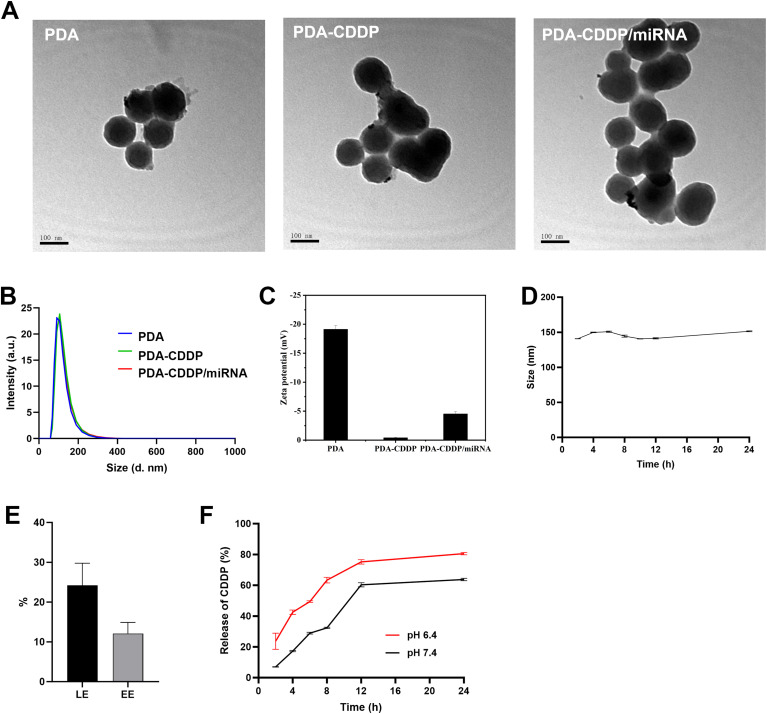
Synthesis and characterization of PDA–CDDP/miRNA. (A) Transmission electron microscopy (TEM) images of PDA, PDA–CDDP, and PDA–CDDP/miRNA nanoparticles. (B) Size distribution of the nanoparticles measured by dynamic light scattering (DLS). (C) Zeta potential measurements of the nanoparticles. (D) Stability of PDA–CDDP/miRNA nanoparticles under physiological conditions. (E) Drug loading capacity (LE) and encapsulation efficiency (EE) of cisplatin (CDDP) within the PDA matrix. (F) CDDP release profile from PDA–CDDP/miRNA nanoparticles.

The synthesis of PDA–CDDP/miRNA nanoparticles was initiated by fabricating monodisperse polydopamine (PDA) nanoparticles through oxidative self-polymerization. Cisplatin (CDDP) and miRNA were then sequentially loaded onto the PDA nanoparticles to form the final PDA–CDDP/miRNA complex. As shown in Fig. 2A, the synthesized PDA nanoparticles exhibited a uniform spherical morphology. Both PDA–CDDP and PDA–CDDP/miRNA maintained similar structural characteristics as the pristine PDA nanoparticles. Dynamic light scattering (DLS) analysis revealed that the hydrodynamic diameters of PDA, PDA–CDDP, and PDA–CDDP/miRNA were comparable (Fig. 2B), suggesting that the particle size remained consistent after modification.

The surface zeta potentials of the nanoparticles were also assessed (Fig. 2C). The zeta potential of PDA was found to be −19.13 ± 0.65 mV, indicating its negatively charged surface. Following CDDP loading, the zeta potential of PDA–CDDP increased to −0.41 ± 0.86 mV, confirming the successful incorporation of cisplatin. Further addition of miRNA led to a slight increase in negative charge, as the negatively charged phosphate backbone of miRNA contributed to a reduction in zeta potential to −4.52 ± 0.41 mV. These results collectively confirm the successful preparation of PDA–CDDP/miRNA nanoparticles.

The stability of PDA–CDDP/miRNA was assessed using DLS in the presence of 5% fetal bovine serum (FBS) (Fig. 2D). The hydrodynamic diameter of PDA–CDDP/miRNA remained stable over 24 hours, indicating that the nanoparticles maintained their structural integrity under physiological conditions. Additionally, the loading efficiency (LE, 24.22 ± 0.06%) and encapsulation efficiency (EE, 12.11 ± 0.03%) of CDDP in the PDA–CDDP nanoparticles were calculated (Fig. 2E and S1†). The CDDP release profile from PDA–CDDP/miRNA demonstrated a pH-dependent release pattern, with a faster release observed under acidic conditions (Fig. 2F). This pH-responsive behavior suggests that the drug will be preferentially released in the acidic tumor microenvironment, potentially minimizing off-target toxicity to normal tissues.

### 3.2 *In vitro* therapeutic efficacy of PDA–CDDP/miRNA

**Fig. 3:**
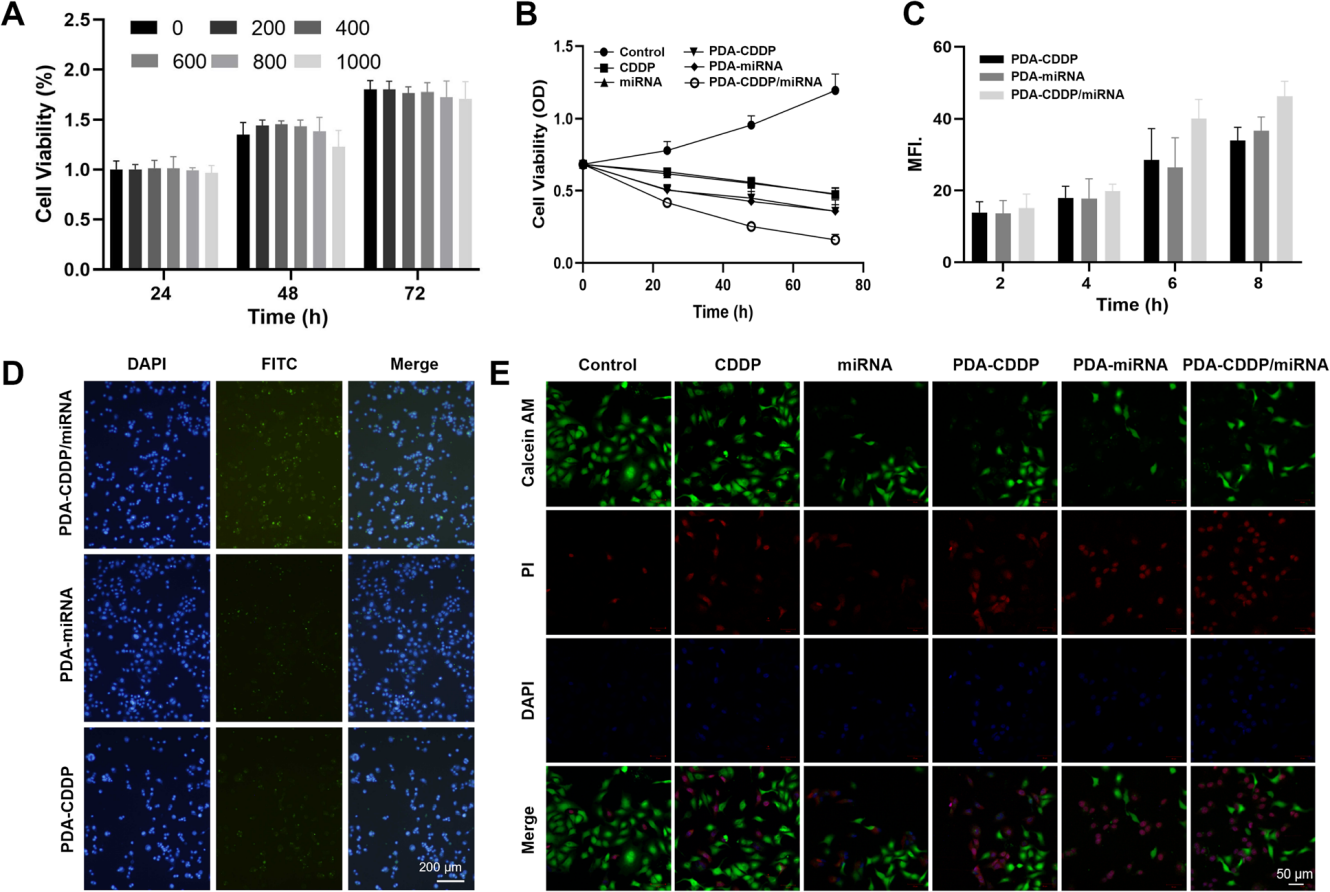
Inhibitory Effects of PDA–CDDP/miRNA on tumor cell a. (A) Cytotoxicity of PDA nanoparticles in 5637 cells. (B) Effects of CDDP, miRNA, PDA–CDDP, PDA–miRNA, and PDA–CDDP/miRNA on the viability of 5637 cells. (C) and (D) Cellular uptake of PDA–CDDP, PDA–miRNA, and PDA–CDDP/miRNA by 5637 cells. (E) Fluorescence images of live/dead staining in 5637 cells treated with CDDP, miRNA, PDA–CDDP, PDA–miRNA, and PDA–CDDP/miRNA.

Prior to combination therapy testing, the cytocompatibility of blank PDA nanoparticles was rigorously quantified using CCK-8 assays (Fig. 3A). The viability of 5637 cells incubated with different concentrations of PDA nanoparticles (0, 200, 400, 600, 800, and 1000 μg mL^−1^) for 24, 48, and 72 hours was evaluated using the CCK-8 assay. Results showed a concentration-dependent cytotoxicity of PDA, with 600 μg mL^−1^ selected as the optimal concentration due to its moderate toxicity profile. The impact of CDDP, miRNA, PDA–CDDP, PDA–miRNA, and PDA–CDDP/miRNA on 5637 cell proliferation was then examined (Fig. 3B). Both CDDP and miRNA individually demonstrated significant inhibition of cell growth. However, the lowest cell viability was observed in cells treated with PDA–CDDP/miRNA, suggesting a synergistic effect between CDDP and miRNA in suppressing tumor growth.

To further explore nanoparticle internalization, PDA was labeled with FITC, and the cellular uptake of PDA–CDDP, PDA–miRNA, and PDA–CDDP/miRNA by 5637 cells was observed (Fig. 3C and D). Fluorescence intensity peaked 8 hours post-incubation, indicating that nanoparticle uptake reached saturation. Additionally, live/dead staining was employed to assess the cytotoxic effects of PDA–CDDP/miRNA on 5637 cells (Fig. 3E). While CDDP alone induced substantial tumor cell killing, miRNA alone exhibited weaker cytotoxicity. Notably, PDA–CDDP/miRNA caused the highest level of cell death, highlighting the enhanced therapeutic efficacy of combining CDDP with miRNA. This synergistic effect underscores the importance of targeting CENPF, which is highly expressed in 5637 cells.

In summary, PDA–CDDP/miRNA significantly inhibited the growth of 5637 bladder cancer cells. The combination of CDDP and miRNA within the PDA nanoparticle framework not only enhanced their individual therapeutic effects but also emphasized the superiority of this targeted approach in suppressing CENPF expression and promoting tumor cell death. These results suggest that PDA–CDDP/miRNA represents a promising strategy for targeted cancer therapy.

### 3.3 *In vivo* antitumor efficacy of PDA–CDDP/miRNA

**Fig. 4:**
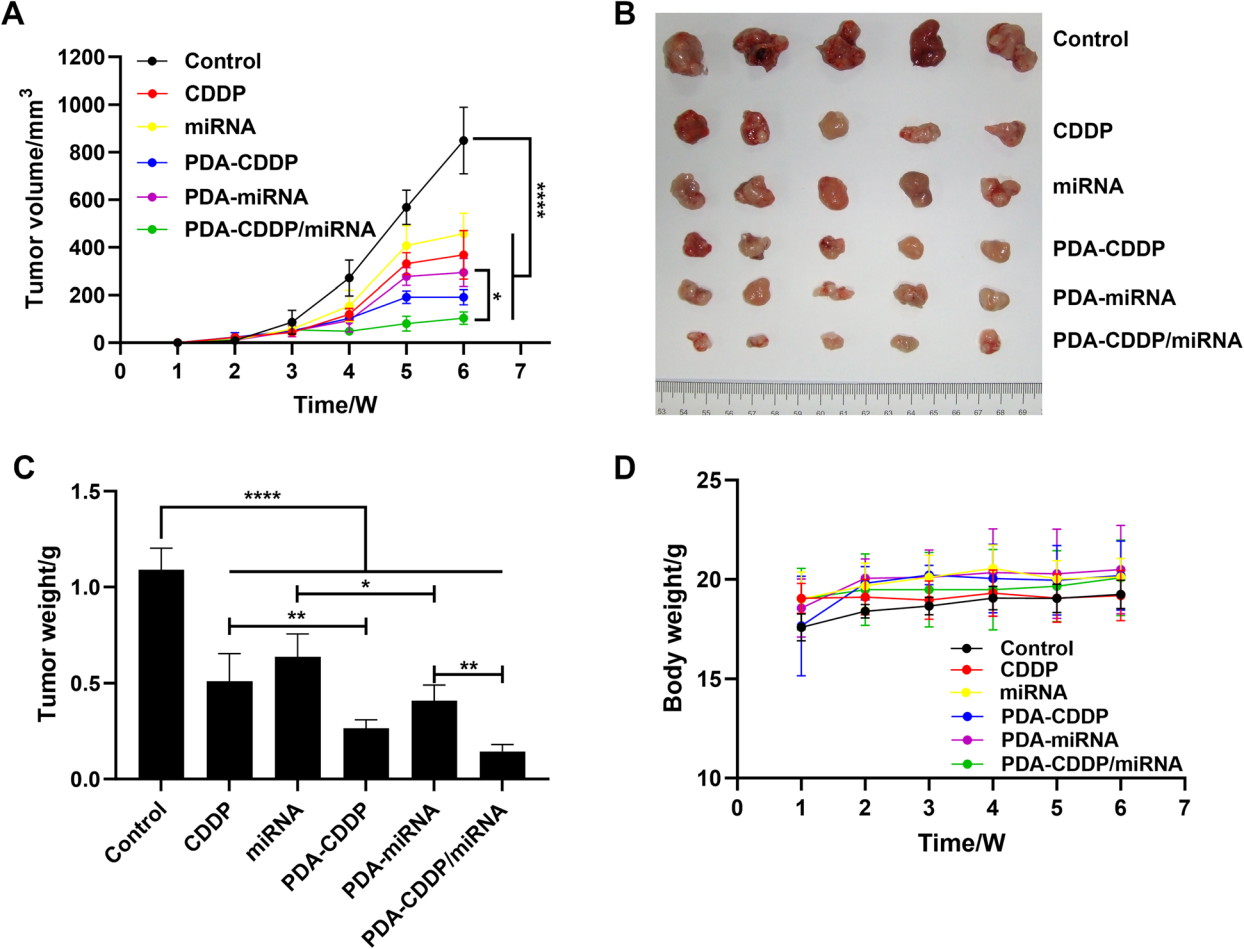
*In Vivo* antitumor efficacy of PDA–CDDP/miRNA. (A) Tumor volume progression curves. (B) Representative images of excised tumors from different treatment groups. (C) Quantitative analysis of tumor weight post-treatment. (D) Body weight dynamics of mice during the treatment period.

To assess the *in vivo* antitumor efficacy of PDA–CDDP/miRNA, a subcutaneous bladder cancer model was established in nude mice. Treatment commenced once the tumors reached the required size. Mice were administered CDDP, miRNA, PDA–CDDP, PDA–miRNA, or PDA–CDDP/miRNA *via* intratumoral injection on days 1, 3, 5, and 7, with the PDA dosage maintained at 5 mg kg^−1^. As shown in Fig. 4A, tumor volume was monitored over a 6-week treatment period. The PDA–CDDP/miRNA group exhibited significant tumor growth inhibition compared to all other groups. Fig. 4B shows representative images of excised tumor tissues at the end of the treatment period, further confirming the potent antitumor effects of PDA–CDDP/miRNA *in vivo*. The corresponding tumor weights, presented in Fig. 4C, highlight a substantial reduction in tumor mass in the PDA–CDDP/miRNA-treated group. Throughout the treatment, no significant changes in body weight were observed across all groups (Fig. 4D), suggesting that the treatments were well-tolerated and did not induce significant toxicity. These findings collectively demonstrate the robust *in vivo* antitumor efficacy of PDA–CDDP/miRNA, underscoring its potential as an effective therapeutic strategy for bladder cancer.

### 3.4 Antitumor mechanisms of PDA–CDDP/miRNA

**Fig. 5:**
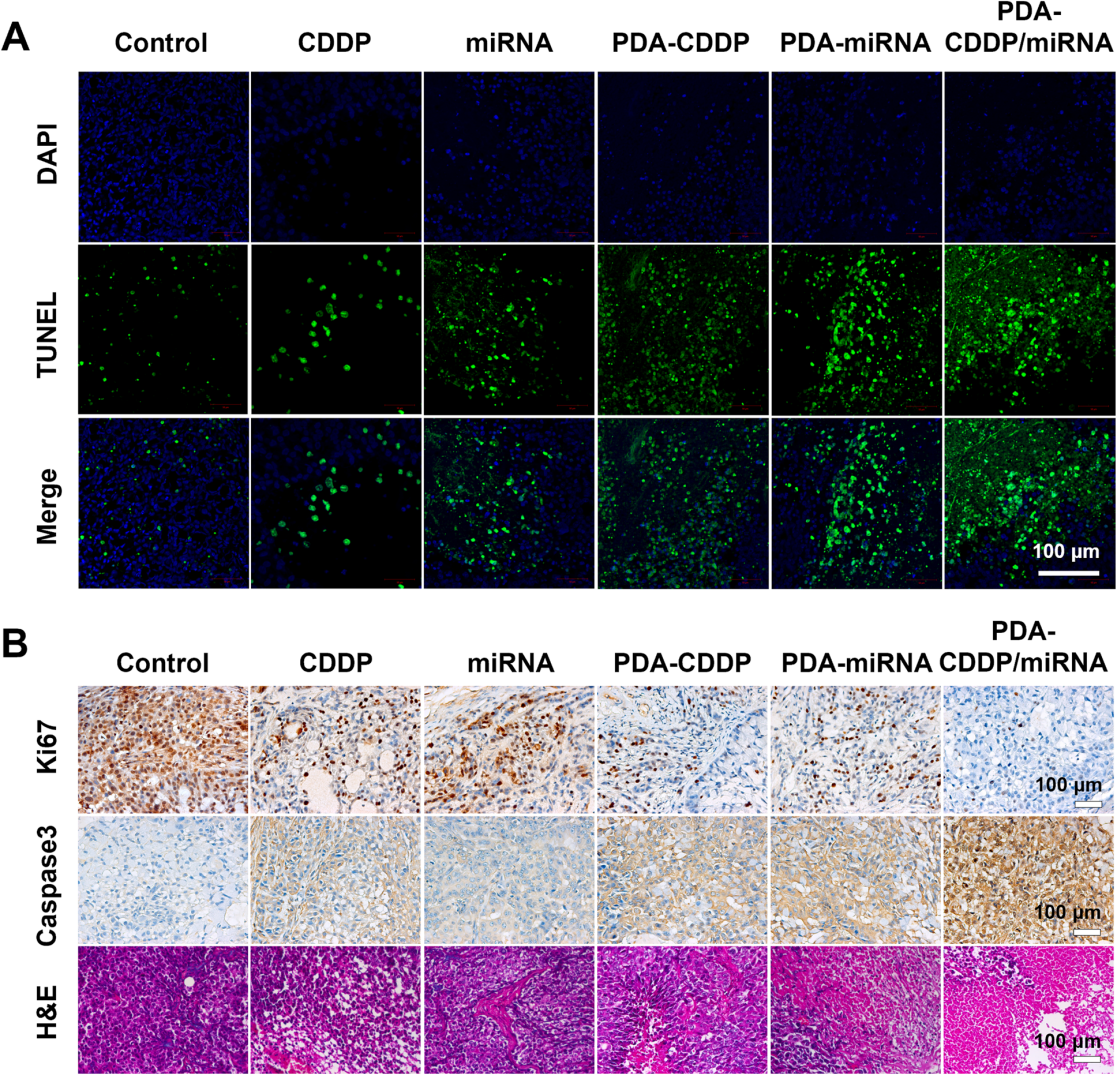
Histopathological analysis of tumor tissues post PDA–CDDP/miRNA treatment. (A) TUNEL immunofluorescence images of tumor tissue sections from different treatment groups. (B) Representative images of Ki67, caspase-3, and H&E staining of tumor tissues.

Cisplatin (CDDP) primarily exerts its antitumor effects by forming DNA adducts, which result in DNA damage. After entering the cell, CDDP undergoes aquation to produce reactive species that create intra- and inter-strand crosslinks with purine bases, especially guanine, in DNA. These crosslinks interfere with DNA replication and transcription, ultimately inhibiting tumor cell proliferation. CENPF, a protein essential for mitosis and cell cycle regulation, is highly expressed in bladder cancer. Suppression of CENPF enhances the efficacy of DNA-damaging agents by making tumor cells more vulnerable to DNA damage during mitosis. To assess the synergistic effects of CDDP and miRNA on DNA damage, we employed TUNEL (terminal deoxynucleotidyl transferase dUTP nick end labeling) staining, which detects DNA strand breaks indicative of apoptosis. TUNEL-positive cells signal apoptosis. As shown in Fig. 5A and S2,† the fluorescence intensity of TUNEL staining was highest in tumor tissues treated with PDA–CDDP/miRNA, exceeding that in tissues treated with CDDP or miRNA alone. This finding suggests that inhibition of CENPF amplifies DNA damage in tumor cells, and the combination of CDDP and miRNA promotes apoptosis.

### 3.5 Detection of PDA–CDDP/miRNA-induced tumor cell apoptosis

Tumor cell apoptosis was further assessed using immunohistochemical staining (Fig. 5B). Ki67 staining indicated a significant reduction in tumor cell proliferation following PDA–CDDP/miRNA treatment. Caspase-3 staining revealed increased apoptosis in tumor cells treated with PDA–CDDP/miRNA. Hematoxylin and eosin (H&E) staining confirmed a marked reduction in tumor tissue size after PDA–CDDP/miRNA treatment. In summary, these findings suggest that PDA–CDDP/miRNA induces significant tumor cell apoptosis, thereby inhibiting the growth of 5637 bladder cancer cells. The combination of CDDP and miRNA within the PDA nanoparticle framework enhances DNA damage and apoptotic signal, demonstrating the therapeutic potential of this approach.

## 4. Conclusions

In this study, we developed a novel nanotherapeutic platform, PDA–CDDP/miRNA, which integrates cisplatin (CDDP) and miR-205-5p within a polydopamine (PDA) nanoparticle system for the targeted treatment of bladder cancer (BCa). The PDA-engineered drug delivery platform demonstrated outstanding biocompatibility and structural stability, complemented by precisely controlled pH-dependent therapeutic release kinetics, which synergistically enhanced neoplasm-specific accumulation while achieving a significant reduction in systemic adverse effects. *In vitro* experiments demonstrated that PDA–CDDP/miRNA significantly inhibited the proliferation of 5637 bladder cancer cells. The combination of CDDP and miR-205-5p showed a synergistic effect in suppressing tumor growth, further confirmed by increased DNA damage and greater induction of apoptosis, as shown by TUNEL, Ki67, and caspase-3 staining. *In vivo* studies in a subcutaneous bladder cancer model confirmed the potent antitumor efficacy of PDA–CDDP/miRNA, with significant tumor growth inhibition and no adverse body weight changes, highlighting its safety and therapeutic potential. Mechanistically, miR-205-5p downregulation of CENPF amplified the DNA-damaging effects of CDDP, disrupting tumor cell division and proliferation. Collectively, these findings underscore the promise of PDA–CDDP/miRNA as a multimodal therapeutic strategy that combines chemotherapy and gene therapy to enhance treatment outcomes for BCa.

## Author contributions

R. X. S.: study design, experimental operation, data analysis, and manuscript writing and revision. X. C.: data analysis. W. T. Z.: study design, manuscript writing, and revision. X. D. Y.: study design, manuscript revision. Z. S. Z.: manuscript revision. H. Q. W.: manuscript revision. G. H. C.: manuscript revision. J. S. X.: manuscript revision. S. X. Z.: manuscript revision. All authors reviewed and approved the final version of the manuscript.

## Conflicts of interest

There are no conflicts to declare.

## Supplementary Material

RA-015-D5RA01513H-s001

## Data Availability

All relevant data are within the manuscript and the data supporting this article have been included as part of the ESI.†
