# Supplementary material for: Nano-delivery of miRNA inhibiting CENPF combined with cisplatin for bladder cancer treatment
Source: RSC Adv. 2025 Jun 13;15(25):20183–91. doi: 10.1039/d5ra01513h (PMC12163902; doi:10.1039/d5ra01513h)
Supplement: RA-015-D5RA01513H-s001 [file RA-015-D5RA01513H-s001.pdf]

Supplementary Information (SI) for RSC Advances.
This journal is © The Royal Society of Chemistry 2025

Electronic Supplementary Information

Nano-delivery of miRNA inhibiting CENPF combined with cisplatin for bladder cancer treatment

Ruixiang Song[a,b], Xin Chen[c], Zhensheng Zhang[b], Huiqing Wang[b], Guanghua Chen[b],Jinshan Xu[b], Shuxiong Zeng[b], Wentao Zhang[a,d*], Xudong Yao[a,d*]

[a]Department of Urology, Shanghai Tenth People's Hospital, Tongji University, Shanghai, 200072, China

[b]Department of Urology, Changhai Hospital, Naval Medical University, Shanghai, 200433, China.

[c]Department of Urology, Zhejiang Qiushi Cardiovascular Hospital,Hangzhou,310011, China

[d]Urologic Cancer Institute, School of Medicine, Tongji University, Shanghai, 200072,China.

*Corresponding author:
zhangwentao98@163.com (W.T.Z.)
yaoxudong1967@163.com (X.D.Y.)

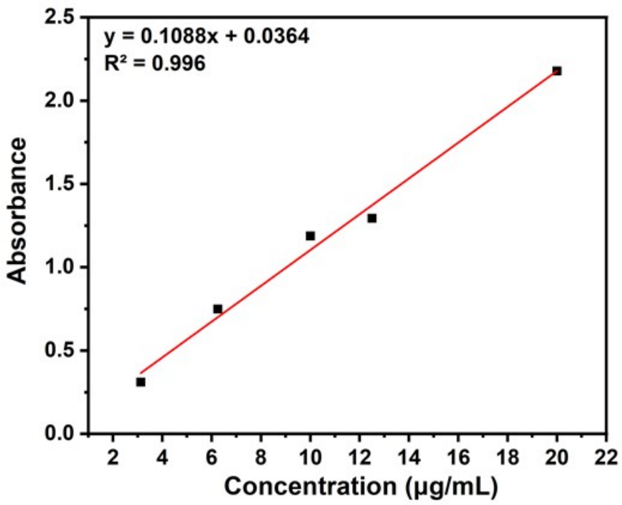

Figure S1. The standard curve of CDDP.

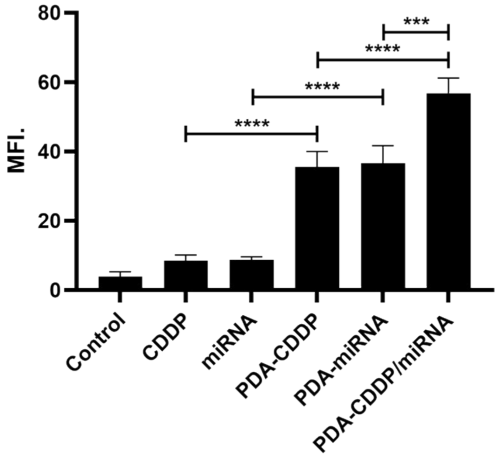

Figure S2. Statistical analysis of TUNEL fluorescence intensity in tumor tissues after different treatments.
